# Short- and Long-term Outcomes of Robotic versus Conventional Minimally Invasive Esophagectomy for Esophageal Cancer: A Multicenter Propensity Score-matched Cohort Study

**DOI:** 10.1245/s10434-026-19091-5

**Published:** 2026-01-22

**Authors:** Shigeru Tsunoda, Hisahiro Hosogi, Shohei Matsufuji, Yukie Yoda, Susumu Shibasaki, Kenoki Ohuchida, Hiroshi Okabe, Tatsuto Nishigori, Seiichiro Kanaya, Hirokazu Noshiro, Koichi Suda, Ichiro Uyama, Kazutaka Obama

**Affiliations:** 1 Department of Surgery, Graduate School of Medicine, Kyoto University, Kyoto, Japan; 2 Department of Surgery, Japanese Red Cross Osaka Hospital, Osaka, Japan; 3 Department of Surgery, Faculty of Medicine, Saga University, Saga, Japan; 4 Department of Surgery, Fujita Health University School of Medicine, Toyoake, Japan; 5 Department of Surgery and Oncology, Graduate School of Medical Sciences, Kyushu University, Fukuoka, Japan; 6 Department of Gastroenterological Surgery, New Tokyo Hospital, Matsudo, Japan; 7 Department of Advanced Robotic and Endoscopic Surgery, Fujita Health University, Toyoake, Japan

**Keywords:** Esophageal cancer, Esophagectomy, Robot, Minimally invasive

## Abstract

**Background:**

Although robot-assisted minimally invasive esophagectomy (RAMIE) reportedly provides better short-term and comparable long-term outcomes compared with open esophagectomy, its long-term outcomes versus those of minimally invasive esophagectomy (MIE) remain insufficiently investigated. This multicenter retrospective cohort study aimed to investigate the perioperative safety, efficacy, and long-term survival of patients of RAMIE versus MIE for esophageal cancer.

**Methods:**

We included patients with cStage 0-IVa thoracic esophageal cancer who underwent esophagectomy through the right thoracic cavity between January 2016 and December 2019 in six Japanese hospitals. The short- and long-term outcomes between RAMIE and MIE were compared by using propensity score matching.

**Results:**

After matching, 268 of 396 patients were analyzed. Compared with MIE, RAMIE had a longer operative time (629 vs. 574 min, *p* < 0.01), a trend toward less severe morbidity (Clavien–Dindo grade ≥ III: 18% vs. 23%), and a lower incidence (22% vs. 34%, *p* = 0.02) and mitigated severity of recurrent laryngeal nerve (RLN) palsy (*p* = 0.040). Blood loss, inhospital mortality, and the mediastinal node harvest were similar between the two techniques. The 3- and 5-year overall survival rates were 77% and 66% for RAMIE and 74% and 66% for MIE (hazard ratio [HR] 0.89; 95% confidence interval [CI] 0.57–1.37; *p* = 0.59). Relapse-free survival was also similar (3-year 64% vs. 63%; 5-year 61% vs. 59%; HR 0.87; 95% CI 0.60–1.28; *p* = 0.49).

**Conclusions:**

RAMIE reduced the incidence and severity of RLN palsy despite requiring a longer operation time and demonstrated similar long-term outcomes to MIE.

The standard treatment for resectable esophageal cancer is esophagectomy plus regional lymphadenectomy with or without preoperative chemotherapy or chemoradiotherapy unless endoscopic treatment is indicated.^1^ However, esophagectomy remains one of the most invasive surgeries for gastrointestinal malignancies, and the postoperative morbidity, including recurrent laryngeal nerve (RLN) palsy, pneumonia, and anastomotic leakage, may ruin patients’ quality of life and sometimes may be fatal. In 1992, a minimally invasive approach was first adopted in esophagectomy.^2^ Since then, conventional open thoracotomy has gradually shifted to thoracoscopy.^3^ However, thoracoscopic esophagectomy, also called minimally invasive esophagectomy (MIE), is a highly technically demanding operation because of the collision of straight forceps with the rib in a narrow intercostal space. One of the solutions for this complexity is robot-assisted minimally invasive esophagectomy (RAMIE) using the da Vinci surgical robotic system.

Although the ROBOT trial has reported that RAMIE is safe and effective, with better short-term and comparable long-term outcomes than open esophagectomy.^4^ Recently, the REVATE trial, a randomized controlled trial comparing RAMIE with MIE, has reported that RAMIE exhibited better short-term outcomes than MIE.^5^ However, the evidence of long-term outcomes of RAMIE compared with MIE remains limited. Therefore, we conducted a multicenter retrospective cohort study to compare the perioperative safety and efficacy, including the long-term survival of patients of RAMIE versus MIE in patients with esophageal cancer.

## Patients and Methods

### Data Collection

This multicenter retrospective cohort study was conducted across six leading hospitals. Since 2009, we have regularly undertaken the FKQ meeting, which includes Fujita University, Kyoto University, Kyusyu (“Q”) University, and their affiliated hospitals, to develop cutting-edge minimally invasive upper gastrointestinal oncological surgery.

In this study, we collected and retrospectively assessed data from the prospectively maintained databases. Specifically, we included patients with cStage 0-IVa histologically proven primary thoracic esophageal cancer who underwent esophagectomy with upper mediastinal dissection through the right thoracic cavity between January 2016 and December 2019. Those with R1/R2 resection, salvage surgery, and two-stage operation were excluded. Given that the Japanese public insurance system started the reimbursement for RAMIE for esophageal cancer in 2018, da Vinci surgical robotic system was only used if the patient agreed with the private practice before the reimbursement.^6–8^ Thereafter, RAMIE was indicated depending on the availability and institutional circumstances. During the study period, all patients eligible for surgery were assigned to either conventional MIE, RAMIE, or mediastinoscopic esophagectomy, and none of them underwent open esophagectomy. The ethics committees of Kyoto University Hospital (R3960), as well as that of each participating hospital, approved this study.

### Surgical Procedures, Perioperative Management, and Follow-up

All patients underwent clinical staging based on the 8^th^ edition of the Union for International Cancer Control staging,^9^ and they individually received multidisciplinary treatment in accordance with the Japanese Guidelines for Diagnosis and Treatment of Carcinoma of the Esophagus.^1,10^ Neoadjuvant chemotherapy with cisplatin plus 5-fluorouracil was generally given for fit patients who had cStage I (T1N1M0) and cStage II or greater disease in accordance with the Japanese guidelines.^11–13^ Neoadjuvant chemoradiotherapy was sometimes considered for patients with concerns about the surgical margin. Board-certified surgeons, who were also qualified by the Endoscopic Surgical Skill Qualification System of the Japanese Society for Endoscopic Surgery, performed all the procedures.^14^ The extent of lymph node dissection was determined according to the Japanese Guidelines for Diagnosis and Treatment of Carcinoma of the Esophagus.^1,10^ The thoracic procedure was performed in the prone position, and the gastric conduit was generally created laparoscopically, including robot assistance. For left upper mediastinal dissection, the azygos arch and right bronchial artery were generally divided, and the dorsal side of the upper esophagus was mobilized from the thoracic duct, provided that the radial margin can be safely secured. The left sympathetic nerves were also commonly preserved. Therefore, the extent of resection was well standardized among the institutions. However, the detailed surgical procedures varied among institutions, particularly regarding the timing of esophageal transection and the identification of the left RLN. Some institutions transected the esophagus before removing the lymphatic tissue from the tracheal wall,^15–17^ whereas others dissected it from the trachea while lifting the esophagus without transection.^18^ With respect to the identification of the left RLN, some institutions identified the nerve early during dorsal mobilization, whereas others identified it ventrally from the esophagus after the dissection between the esophagus and the membranous trachea.^16–19^ The reconstruction route was selected at the surgeon’s discretion. Postoperative management was conducted as per the standardized institutional protocol. Patients were generally followed up every 3–6 months with chest and abdominal computed tomography scans and blood tests, and underwent upper endoscopy every 6–12 months for the first 3 years. After 3 years, follow-up was continued every 6 months with blood tests, upper endoscopy, and chest and abdominal computed tomography scans until 5 years postoperatively.

### Outcomes

Adverse events occurring within 30 days postoperatively or during the inhospital period indicated postoperative complications, and severity was evaluated using the Clavien–Dindo (C–D) classification (grades I–V).^20^ Grade II or higher indicated the presence of complications, and complications graded as III or higher were considered severe. Overall survival (OS) was assessed from the date of esophagectomy to the date of last follow-up or death, whichever occurred first. Relapse-free survival (RFS) was measured from the esophagectomy date to the date of the initial occurrence of relapse or death from any cause. Short-term outcomes were also compared during and after the learning curve period (<27 cases and ≥27 cases).^7^ In all hospitals, postoperative laryngoscopy was routinely performed to assess RLN palsy. The site of the first recurrence could either be local (regional cervical/mediastinal/abdominal lymph nodes and anastomotic site), distant (hematological metastasis, pleural/peritoneal/pericardial dissemination, nonregional lymph nodes), or distant + local (combination of distant and local).

### Statistical Analysis

Individual propensity scores were calculated using logistic regression analysis according to the following 15 variables: age, sex, body mass index, American Society of Anesthesiologists Physical Status, preoperative hemodialysis, systemic steroid use, obstructive pulmonary disease, histology, tumor location, clinical T, N, M classification, preoperative therapy, lymphadenectomy field, and organ used for reconstruction. Subsequently, we conducted a 1:1 propensity score-matching analysis to compare the short-term outcomes between RAMIE and MIE, using nearest-neighbor matching (caliper size = 0.2). We employed the chi-square test or Fischer’s exact test for comparing categorical variables as appropriate, and the nonparametric Mann-Whitney *U* test for comparing continuous variables. Survival rates were estimated using the Kaplan-Meier method and compared with the log-rank test. A *p*-value below 0.05 was considered statistically significant. Statistical data were analyzed by using JMP Pro 17.0.0 software (SAS Institute, Cary, NC) and R version 3.6.2 software (Vienna, Austria).

## Results

### Patient Characteristics

**Fig. 1:**
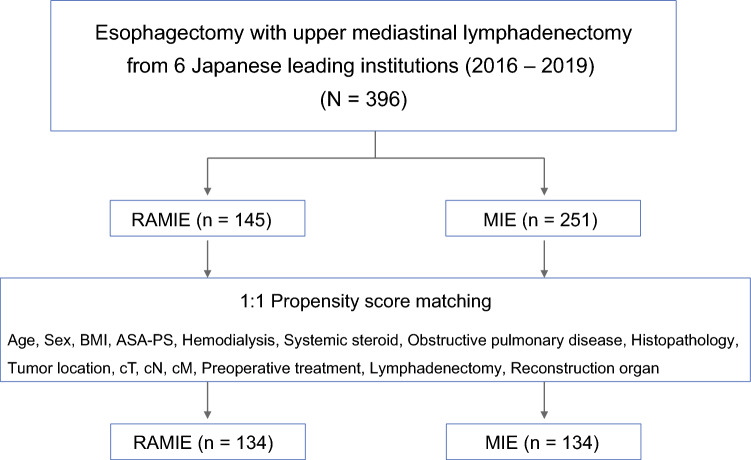
Patient flow diagram

We included 396 patients (RAMIE, 145; MIE, 251) who underwent esophagectomy between 2016 and 2019 at six institutions (Fig. 1). Table 1 summarizes their baseline characteristics. After matching, 134 patients were matched for each group, and the demographic factors and preoperative oncological characteristics were well balanced between the two groups.

**Table 1:** Baseline demographics of the patients before and after matching

	Before matching (N = 396)			After matching (N = 268)		
Variables	RAMIE (n = 145)	MIE (n = 251)	SMD	RAMIE (n = 134)	MIE (n = 134)	SMD
Median age, IQR (yr)	67 (60–74)	69 (63–74)	0.149	67 (60–73)	69 (63–74)	0.105
Male/female ratio	111/34	208/43	0.158	106/28	104/30	0.036
Median body mass index, IQR (kg/m^2^)	21.8 (19.6–23.6)	21.5 (19.3–23.8)	0.030	21.8 (19.6–23.7)	21.5 (19.7–23.8)	0.001
ASA class			0.081			0.098
1	36 (25%)	63 (25%)		34 (25%)	36 (27%)	
2	106 (73%)	172 (69%)		97 (72%)	93 (69%)	
3	3 (2%)	16 (6%)		3 (2%)	5 (4%)	
Systemic use of steroid	4 (3%)	4 (2%)	0.080	3 (2%)	4 (3%)	0.047
Preoperative hemodialysis	0 (0%)	2 (1%)	0.127	0 (0%)	0 (0%)	<0.001
Obstructive pulmonary disease	34 (23%)	52 (21%)	0.066	30 (22%)	33 (25%)	0.053
Histopathology			0.082			<0.001
Squamous cell carcinoma^a^	136 (94%)	240 (96%)		129 (96%)	129 (96%)	
Other	9 (6%)	11 (4%)		5 (4%)	5 (4%)	
Adenocarcinoma	7	10		4	4	
Carcinosarcoma	1	1		1	1	
Melanoma	1					
Tumor location			0.267			0.054
Upper	20 (14%)	25 (10%)		17 (13%)	15 (11%)	
Middle	76 (52%)	109 (43%)		72 (54%)	75 (56%)	
Lower	49 (34%)	117 (47%)		45 (34%)	44 (33%)	
Preoperative therapy			0.150			0.050
Upfront surgery	65 (45%)	94 (37%)		60 (45%)	62 (46%)	
Neoadjuvant chemotherapy	73 (50%)	143 (57%)		67 (50%)	64 (48%)	
Neoadjuvant chemoradiotherapy	7 (5%)	14 (6%)		7 (5%)	8 (6%)	
Clinical T stage			0.025			<0.001
1	60 (41%)	107 (43%)		59 (44%)	59 (44%)	
1a	5	18		5	12	
1b	55	89		54	47	
2-4	85 (59%)	144 (57%)		75 (56%)	75 (56%)	
2	26	37		23	21	
3	53	97		50	46	
4a	6	10		6	6	
Clinical N stage			0.097			0.060
0	70 (48%)	109 (43%)		65 (49%)	69 (51%)	
1-3	75 (52%)	142 (57%)		69 (51%)	65 (49%)	
1	45	97		43	45	
2	24	42		20	19	
3	6	3		6	1	
Clinical M stage			0.156			0.088
0	138 (95%)	246 (98%)		131 (98%)	129 (96%)	
1	7 (5%)	5 (2%)		3 (2%)	5 (4%)	
Clinical TNM stage			0.004			0.071
I	56 (39%)	97 (39%)		55 (41%)	52 (39%)	
II	34 (23%)	47 (19%)		30 (22%)	30 (22%)	
III	36 (25%)	87 (35%)		34 (26%)	38 (28%)	
IV	19 (13%)	20 (8%)		15 (11%)	14 (10%)	
Lymphadenectomy			0.022			0.015
Two field	68 (47%)	115 (46%)		62 (46%)	61 (45%)	
Three field	77 (53%)	136 (54%)		72 (54%)	73 (55%)	
Reconstruction			0.012			<0.001
Gastric conduit	139 (96%)	240(96%)		128 (96%)	128 (96%)	
Other	6 (4%)	11 (4%)		6 (4%)	6 (4%)	
Jejunum	3	5		3	4	
Colon	3	6		3	2	
Abdominal procedure			0.122			0.174
Minimally invasive	136 (94%)	242 (96%)		125 (93%)	130 (97%)	
Laparoscopic/robot	103/33	242/0		97/28	130/0	
Open	9 (6%)	9 (4%)		9 (7%)	4 (3%)	

*ASA* American Association of Anesthesiologists; *SMD* standardized mean difference

^a^Including adenosquamous cell carcinoma and basaloid carcinoma

### Short-term Outcomes

**Fig. 2:**
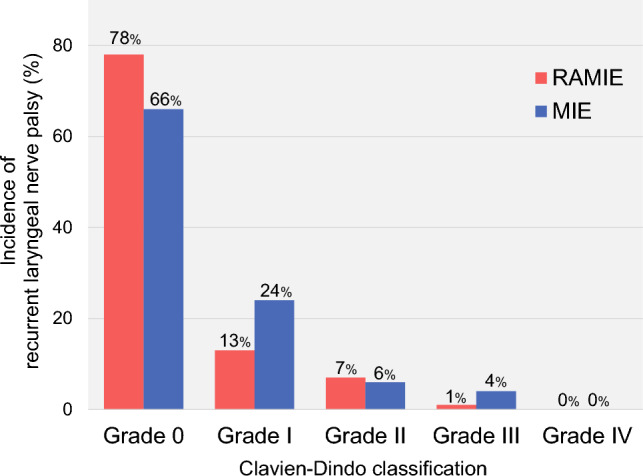
Incidence of recurrent laryngeal nerve palsy (%) according to the Clavien–Dindo classification. Fisher’s exact test revealed a statistically significant difference in the distribution of grades between RAMIE and MIE (*p* = 0.040)

Table 2 lists the short-term outcomes of the matched cohort. Although the total and the thoracic operating time were significantly longer in RAMIE than in MIE (629 vs. 574 min, *p* = 0.002, 298 vs. 246 min, *p* < 0.001, respectively), the amount of blood loss (88 vs. 78 g) and in-hospital mortality (0.8% vs. 0.8%) were comparable between these groups. The RAMIE group also showed a trend of lesser severe postoperative morbidity (Clavien-Dindo grade ≥III) (18% vs. 23%) and a significant reduction of RLN palsy compared with MIE (22% vs. 34%, *p* = 0.02). Figure 2 shows the details of RLN palsy according to the Clavien–Dindo classification. Notably, RAMIE significantly reduced the severity of RLN palsy compared with MIE (*p* = 0.030). Regarding oncological outcomes, the number of harvested mediastinal nodes (24 vs. 26) and the number of harvested left upper mediastinal nodes (106recL, 106tbL, and 101L; 6 vs. 6) were equipoise between two groups.

**Table 2:** Short-term outcomes of the patients after matching

Variables	RAMIE (n = 134)	MIE (n = 134)	*p-value*
Total operating time, IQR (min)	629 (541–716)	574 (505–678)	0.002
Thoracic operating time, IQR (min)	298 (249–360)	246 (213–294)	<0.001
Intraoperative blood loss, IQR (mL)	88 (40–156)	78 (32–150)	0.594
Conversion to conventional MIE/open thoracotomy	0 (0%)	0 (0%)	1.000
Median number of resected total lymph nodes, IQR	54 (41–69)	57 (44–72)	0.451
Median number of resected thoracic lymph nodes, IQR	24 (18-38)	26 (19-34)	0.825
Median number of resected left upper mediastinal lymph nodes, IQR	6 (3–9)	6 (4–9)	0.595
Pathological T stage			0.351
0	2 (1%)	5 (4%)	
1a	22 (16%)	22 (16%)	
1b	55 (41%)	43 (32%)	
2	14 (10%)	19 (14%)	
3	41 (31%)	43 (32%)	
4a	0 (0%)	2 (1%)	
Pathological N stage			0.257
0	74 (55%)	78 (58%)	
1	31 (23%)	28 (21%)	
2	17 (13%)	23 (17%)	
3	12 (9%)	5 (4%)	
Pathological M stage			1.000
0	126 (94%)	126 (94%)	
1	8 (6%)	8 (6%)	
Pathological TNM stage			0.435
0	2 (1%)	3 (2%)	
I	54 (40%)	48 (36%)	
II	36 (27%)	34 (25%)	
III	25 (19%)	37 (28%)	
IV	17 (13%)	12 (9%)	
In hospital mortality	1 (0.8%)	1 (0.8%)	1.000
Severe postoperative morbidity (≥ Grade III)	24 (18%)	31 (23%)	0.364
Overall postoperative morbidity (≥ Grade II)	65 (49%)	72 (54%)	0.464
Anastomotic leakage (≥ Grade II)	14 (10%)	17 (13%)	0.703
Pneumonia (≥ Grade II)	25 (19%)	23 (17%)	0.874
Pulmonary complications other than pneumonia (≥ Grade II)	17 (13%)	9 (7%)	0.105
Atrial fibrillation (≥ Grade II)	6 (4%)	2 (1%)	0.174
Thromboembolism (≥ Grade II)	5 (4%)	1 (1%)	0.122
Chylothorax (≥ Grade II)	5 (4%)	2 (1%)	0.284
Recurrent laryngeal nerve palsy (any grade)	29 (22%)	46 (34%)	0.022

### Learning Curve of RAMIE

When comparing during and after the learning curve (<27 cases and ≥27 cases), the total and the thoracic operating time were significantly decreased from 677 to 605 min, and 349 to 273 min, respectively. The number of resected total, thoracic, and left upper mediastinal nodes also significantly increased, but the overall RLN palsy remained stable (21% and 22%). However, the incidence of severe postoperative morbidity increased from 9% to 25%, suggesting that careful and conservative lymphadenectomy was performed during the learning curve period (Table 3).

**Table 3:** Short-term outcomes of the patients of RAMIE during (<27 cases) and after (≥27 cases) the learning curve

Variables	During the learning curve (<27 cases) (n = 58)	After the learning curve (≥27 cases) (n = 76)	*p-value*
Total operating time, IQR (min)	677 (538–747)	605 (542–691)	0.032
Thoracic operating time, IQR (min)	349 (254–399)	273 (244–315)	<0.001
Intraoperative blood loss, IQR (mL)	81 (32–140)	90 (40–190)	0.081
Conversion to conventional MIE/open thoracotomy	0 (0%)	0 (0%)	1.000
Median number of resected total lymph nodes, IQR	50 (39–65)	55 (45–72)	0.039
Median number of resected thoracic lymph nodes, IQR	21 (17-28)	28 (20-50)	<0.001
Median number of resected left upper mediastinal lymph nodes, IQR	6 (2–8)	6 (4–10)	0.017
In hospital mortality	1 (1.7%)	0 (0%)	0.433
Severe postoperative morbidity (≥ Grade III)	5 (9%)	19 (25%)	0.022
Overall postoperative morbidity (≥ Grade II)	26 (45%)	39 (51%)	0.489
Recurrent laryngeal nerve palsy (any grade)	12 (21%)	17 (22%)	0.836

### Long-term Outcomes

**Fig. 3:**
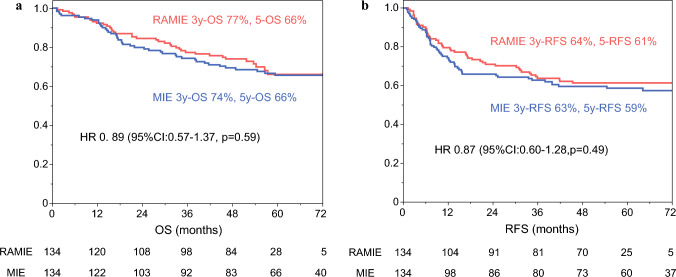
Kaplan–Meier survival curves of the overall survival (OS) (**A**) and relapse-free survival (RFS) (**B**) for patients who underwent RAMIE (red) and MIE (blue)

The 3-year OS was 77% in the RAMIE group and 74% in the MIE group, whereas the 5-year OS was 66% in both groups (hazard ratio [HR] 0.89; 95% confidence interval [CI] 0.57, 1.37, *p* = 0.59] (Fig. 3A), with a median follow-up period of 53 months. Moreover, the RAMIE and MIE groups obtained 64% and 63% for the 3-year RFS, and 61% and 59% for the 5-year RFS, respectively (HR 0.87; 95% CI 0.60, 1.28, *p* = 0.49) (Fig. 3B).

**Fig. 4:**
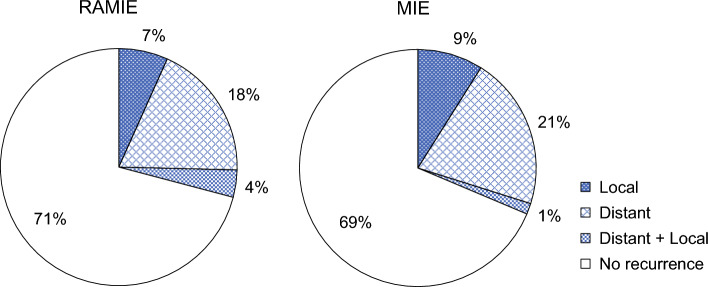
The site of the first recurrence after RAMIE or MIE

Postoperative recurrence was noted in 39 (29%) and 42 (31%) patients in the RAMIE and MIE groups, respectively. Local recurrence was less prevalent in the RAMIE group than in the MIE group (9 [7%] vs. 12 [9%] patients), although the difference did not reach statistical significance. Distant recurrence, including the combination with local recurrence, was observed in 30 patients (22%) of both groups (Fig. 4).

## Discussion

In this study, we retrospectively evaluated and compared the short-term and long-term outcomes between RAMIE and MIE via propensity score matching. This study is the first to compare long-term outcomes between such methods in Japan, where intensive upper mediastinal lymphadenectomy up to the cervicothoracic border is routinely performed.^15,17,21–23^ This study showed similar short-term and long-term outcomes in both methods. However, as expected, RLN palsy was significantly mitigated in the RAMIE group, although the operating time was 1 hr longer. Interestingly, our data suggested the superiority of the robotic upper mediastinal dissection in RLN palsy reduction but not in the number of dissected nodes. This finding may be explained by our matured concept of the lymph node dissection and the extent of the lymphadenectomy being consistent in both methods.^15,16,18^ Indeed, the RAMIE and MIE groups in our study had 24 and 26 harvested mediastinal nodes, while 16 and 14 in the REVATE trial.^5^ The cause of postoperative RLN palsy after esophagectomy is categorized as direct mechanical trauma (e.g., thermal injury) and indirect injury (e.g., excessive traction). From a surgical point of view, articulating tremorless robotic forceps without interference from the ribs is highly beneficial for gentle dissection along the RLNs.

The longer operation time in RAMIE may be explained by the following points. First, all participating surgeons were less experienced in performing RAMIE versus MIE. Indeed, 43% of RAMIE was performed before the 27 case experience that we reported our learning curve,^7^ and a significant reduction in total operation time was observed from 677 min to 605 min after the learning curve period. Second, more precise robotic dissection with monopolar or bipolar diathermy may prolong the procedure time than advanced energy devices, including ultrasonically activated devices or vessel-sealing systems in conventional MIE. Although Vessel Sealer Extend, a bipolar device for vessel sealing and cutting, is commonly used in the middle and lower mediastinum in RAMIE, its bulky size and lateral thermal spread limit its use around the RLN. However, the difference would be shortened in a certain period, as demonstrated by the RAMIE trial from high-volume centers in China,^24^ which required a minimum of 40 case experience. As the learning curve in this study (27 cases) was determined based on operative time, it does not necessarily reflect the incidence of RLN palsy. Indeed, the comparison of short-term outcomes between the early phase (<27 cases) and the late phase (≥27 cases) of the learning curve showed a significant reduction in operative time, accompanied by an increased lymph node yield in the total, thoracic, and left upper mediastinal fields (Table 3). However, both the incidence and severity of RLN palsy remained stable before and after the learning curve and were lower than those observed with MIE (data not shown).

Internationally, our operative time—comparable to the Japanese RAMIE standard (565 min)—remained relatively long even beyond the learning curve.^25^ In contrast, operative times reported in the REVATE trial^5^ and UGIRA registry^26^ were approximately 300–400 min, with a total lymph node yield of 26–34. In this study, however, a median of 54 lymph nodes was harvested, suggesting that the prolonged operative time reflects the meticulous and extensive lymphadenectomy performed.

Regarding the long-term outcomes, the Kaplan-Meier curve of RAMIE was consistently above that of MIE, but the difference was not statistically significant. As the intensity of the lymphadenectomy was similar in both the minimally invasive approaches, the survival benefit was not significant. However, slightly better survival could reflect the trend of lesser postoperative morbidity.

As mentioned, this study was from one of the leading groups of minimally invasive/robotic surgery in Japan, applying robotic gastrointestinal surgery since the early 2010s and conducting the clinical trial approved for Advanced Medical Technology (“Senshiniryo”) B by the Ministry of Health, Labour, and Welfare for future coverage under the universal medical insurance program.^27^ However, this study included the data of early cases of RAMIE. Our study period (2017–2019) was the exact time da Vinci surgical robotic system Xi was introduced and RAMIE explosively increased because of Japanese health insurance reinforcement in 2018. Therefore, the matured RAMIE currently being performed may be superior to our study results.

The present study has some limitations. First, this study was retrospective in design; thus, unmeasured confounding factors were inevitable. The indication for RAMIE prior to public health insurance approval was solely dependent on whether or not the patient could afford it. Second, it was conducted by one of the leading hospitals for robotic surgery, which have been dedicated to performing meticulous and extensive lymph node dissection. Moreover, the standard treatment for esophageal cancer differs between Japan and Western countries. In Japan, where squamous cell carcinoma predominates, neoadjuvant chemotherapy followed by esophagectomy with radical mediastinal lymph node dissection is standard. In contrast, in Western countries, where adenocarcinoma is more common, preoperative chemoradiotherapy with limited mediastinal dissection is a widely accepted treatment strategy as well as neoadjuvant chemotherapy. Therefore, these results may not be directly generalizable to Western clinical practice. Third, R0 resection rate was not compared in this study, as we only included complete resection cases with mandatory upper mediastinal dissection, which is usually omitted if a noncurative resection becomes apparent during surgery. Fourth, it could not evaluate the socioeconomic factors. Further studies with larger sample sizes are warranted to establish the benefits of RAMIE.

## Conclusion

RAMIE proved to be a safer and more feasible procedure when performed by experienced surgeons. Despite the 1-hr longer operation time, it reduced the incidence and severity of RLN palsy compared to conventional MIE. The long-term outcomes of RAMIE were at least equipoise to the conventional MIE.
